# Pea plants conditionally sanction less effectively fixing rhizobia at the level of whole nodules rather than single cells

**DOI:** 10.1093/jxb/erag191

**Published:** 2026-04-27

**Authors:** Thomas J Underwood, Beatriz Jorrin, Lindsay A Turnbull, Philip S Poole

**Affiliations:** Department of Biology, University of Oxford, Oxford OX1 3EL, UK; Department of Biology, University of Oxford, Oxford OX1 3EL, UK; Department of Biology, University of Oxford, Oxford OX1 3EL, UK; Department of Biology, University of Oxford, Oxford OX1 3EL, UK; University of Warwick, UK

**Keywords:** Conditional sanctioning, legumes, mixed nodules, rhizobia, sanctioning

## Abstract

Legumes sanction root nodules containing rhizobial strains with low nitrogen fixation rates (less effectively fixing). Pea (*Pisum sativum*) nodules contain both undifferentiated bacteria and terminally differentiated nitrogen-fixing bacteroids. It is critical to understand how sanctions act on both bacteria and bacteroids, and how they differ. In addition, less effective strains could potentially evade sanctioning by entering the same nodule as an effective strain (i.e. piggybacking). *Pisum sativum* was co-inoculated with pairwise combinations of three strains of rhizobia with different effectiveness, to test whether ineffective strains can evade sanctions in this way. We assessed the effect of sanctions on nodule populations of bacteria and bacteroids using flow cytometry and the effects on nodule internal structure using confocal microscopy. We show that sanctioning lowered bacteroid populations and caused a reduction in the size of bacteria. Sanctions also precipitated an early change in nodule cell morphology. In nodules containing two strains that differed in their nitrogen-fixation ability, both were treated equally. Thus, peas sanction whole nodules based on their nitrogen output, but do not sanction at the cellular level. Our results demonstrate that peas conditionally sanction at the whole-nodule level, providing stability to the symbiosis by reducing the fitness of ineffective strains, but cannot target individual strains in a mixed nodule.

## Teaser

Host sanctions result in the collapse of bacteroid populations and lead to premature nodule senescence. Peas cannot selectively punish strains within mixed nodules, instead sanctioning the whole nodule.

## Introduction

Legumes have overcome nitrogen limitation by establishing a mutually beneficial interaction with nitrogen-fixing bacteria, hosted within nodules along their root systems (Poole *et al*., 2018). Certain species of legumes, such as peas, form nodules in which some bacteria undergo terminal differentiation to become large swollen cells called bacteroids, which carry out the costly process of nitrogen fixation, but cannot resume their free-living existence (Mergaert *et al*., 2006). The plant provides carbon compounds in the form of photosynthetically derived dicarboxylates in return for ammonia provided by the bacteroids, and this relationship continues until the nitrogen requirements of the plant have been met (Udvardi and Poole, 2013). At this point, nodule senescence ensues and, while the bacteroids then die (Denison, 2000), any undifferentiated bacteria are presumably released into the soil (Timmers *et al*., 2000). The presence of a host legume, specifically clover rather than pea, has been shown to increase the soil population of rhizobia (Beringer *et al*., 1979); therefore, by engaging in the symbiosis, legumes potentially provide a significant fitness advantage to rhizobia.

However, this interaction presents an evolutionary dilemma. A ‘cheating’ strain that reduces investment in the costly process of nitrogen fixation might be able to produce more reproductive bacteria within the nodules, potentially giving it a fitness advantage. Legumes are known to have evolved sanctions in order to maximize investment in cooperative bacteria, with the consequence of reducing the fitness of cheating bacteria (West *et al*., 2002; Kiers *et al*., 2003; Oono *et al*., 2011; Westhoek *et al*., 2017), and these sanctions can be applied conditionally, with pea plants known to sanction nodules that contain an intermediate-fixing strain only when a better strain is available (Westhoek *et al*., 2021). Intriguingly, while Westhoek *et al*. (2021) did see a significant drop in carbon transport and nodule size by 28 days post-inoculation (dpi), they did not see a significant decrease in the number of viable bacteria within the sanctioned nodule (although the number of viable bacteria had plummeted dramatically by 56 dpi). As such, the cause of the difference in nodule size at 28 dpi, while hypothesized to be due to a significantly smaller bacteroid population, has yet to be proven.

Poorly fixing strains might be able to evade plant sanctions by ‘piggybacking’ on a more effective strain, since multiple strains can occupy a single nodule, at frequencies of >20% (Mendoza-Suárez *et al*., 2020). Notably individual cells of ‘mixed’ nodules only contain one bacterial strain and mixed nodules are ‘sectored’, with separate areas of the mixed nodule containing each strain (Daubech *et al*., 2017). If plants distinguished between cells containing different strains they could prevent piggybacking by sanctioning cells containing the less effective strain. Despite some evidence for so-called cell-autonomous sanctioning, in the form of premature senescence of cells containing an ineffective strain (Regus *et al*., 2014; Daubech *et al*., 2017), other studies, focusing on metabolic evidence, did not show a significant difference in the metabolic profile of cells containing fixing or non-fixing bacteria (Agtuca *et al*., 2020). Crucially the three studies referenced here (Daubech *et al*., 2017; Regus *et al*., 2017; Agtuca *et al*., 2020) were all conducted with different model systems. Thus the presence or absence of cell-autonomous sanctioning may not be consistent across the legumes. None of these studies was carried out on the *Pisum sativum*—*Rhizobium leguminosarum* system. Therefore, this work will expand our current understanding of mixed nodule treatment in this symbiosis.

This study builds on previous work carried out by Westhoek *et al*. (2021) using pea plants and a set of otherwise isogenic rhizobial strains that differ in their ability to fix nitrogen. First, with nodules infected with a single rhizobial strain, we used flow cytometry to measure fitness characteristics of both undifferentiated bacteria and nitrogen-fixing bacteroids; we then used fluorescence microscopy to establish how sanctions influenced the health and morphology of the infected plant cells within those nodules. Second, we used the same techniques on mixed nodules to determine whether or not pea plants carry out cell-autonomous sanctioning within nodules. Our results show that sanctions reduce the number of bacteroids within whole nodules as well as reducing the size of the undifferentiated bacteria. Fluorescence microscopy showed that the breakdown of cells within the nodule occurred prematurely in sanctioned nodules. Our results also show that these sanctions operate at the level of the whole nodule but not at the cellular level. As such, a piggybacking strain cannot be punished independently of the non-cheater within a mixed nodule; however, mixed nodules were treated as ineffective and sanctioned at the nodule level. Therefore, piggybacking is not a particularly effective route for ineffective ‘cheating’ strains. This is because if the ineffective strains make up a significant proportion of bacteroids inside a nodule they will cause whole-nodule sanctioning.

## Materials and methods

### Bacterial strains and culture conditions

Rhizobial strains used in this study are all derivatives of a highly effective nitrogen-fixing strain, *R. leguminosarum* bv. (Rlv) 3841, that infects pea (*P. sativum* L. cv. Avola) (Johnston and Beringer, 1975). The mutant strains thus differ in their nitrogen-fixation ability but are otherwise genetically identical (Table 1). The fixation ability of each of these strains was measured by Westhoek *et al*. (2021) as the ratio of the rate for wild-type *R. leguminosarum* 3841 (Fix^+^). The Fix^−^ strain does not fix nitrogen at all because of a spectinomycin cassette insertion within the gene *nif*H which encodes a core subunit of the nitrogenase enzyme. The Fix^int^ strain fixes at ∼30% that of Fix^+^ due to a spectinomycin cassette insertion in the promoter region of *nifA*, the major transcription factor gene for nitrogen fixation, which throttles its expression. Strains are labelled with a fluorescent marker [mCherry or green fluorescent protein (GFP)] in order to distinguish the nodules formed by each strain (Table 1). Strains were maintained on tryptone-yeast (TY) agar (Beringer, 1974) with the appropriate concentrations of antibiotics (Table 1). For long-term storage, strains were kept at −80 °C in TY with 15–20% glycerol. Rhizobial inoculant was grown on a TY agar slope, and the number of bacteria on the slope was determined by measuring the OD_600_ of the washed slope using a Genesys 150 UV-Visible spectrophotometer. Cells were diluted to ∼5 ×10^7^ ml^−1^.

**Table 1.** Rhizobial strains and their fixation abilities

Name	Strain	Antibiotic resistance	Description	Reference
Fix^+^	Rlv3841	Streptomycin	Rlv3841	Johnston and Beringer (1975)
Fix^+^ mCherry	OPS1341	Streptomycin, gentamicin	Rlv3841 Tn*7*-Gm-mCherry	Westhoek *et al*. (2021)
Fix^+^ GFP	OPS1339	Streptomycin, gentamicin	Rlv3841 Tn*7*-Gm-GFP	Westhoek *et al*. (2021)
Fix^−^–GFP	OPS2270	Streptomycin, spectinomycin, gentamicin	Ω*nifH* Tn*7*-Gm-sfGFP (Rlv3841 mutant, ΩSpc cassette in *nifH*)	Westhoek *et al*. (2021)
Fix^int^–GFP	OPS2268	Streptomycin, spectinomycin, gentamicin	Fix^int^ Tn*7*-Gm-sfGFP (Rlv3841 mutant, ΩSpc cassette in promoter region of *nifA*)	Westhoek *et al*. (2021)
Fix^int^–mCherry	OPS2269	Streptomycin, spectinomycin, gentamicin	Fix^int^ Tn*7*-Gm-mCherry False colour: orange	Westhoek *et al*. (2021)

All strains were derived from Rlv3841 and are provided with a strain code, resistance markers, a short description, and reference.

### Plant growth

Before sowing, all pea seeds were surface-sterilized (1 min in 95% ethanol followed by 5 min in 20% NaClO), rinsed, and left to germinate on 1% w/v agar plates at room temperature in the dark. Seedlings were transplanted after 5 d by transferring them to sterilized 1 litre Azlon beakers containing a 1:1 mixture of silver sand and fine vermiculite, 150 ml of sterilized nitrogen-free nutrient solution, and a 1:1 ratio of the two rhizobial strains (∼0.25×10^7^ cells) (see also Westhoek *et al*., 2017). Beakers were covered with clingfilm to reduce aerial contamination, which was slit after a few days to allow seedlings to grow through. Plants were grown in a growth room (21 °C, 16 h photoperiod) for 28–42 d and watered as necessary from 7 d onwards.

### Bacterial inoculation of plants

Plants were inoculated with either single strains or three pairs of otherwise isogenic strains, differing only in their ability to fix nitrogen. The strains are: wild type or good fixer (Fix^+^); intermediate fixer (Fix^int^); and non-fixer (Fix^−^). This gives three pairwise combinations of a more effective (E) and a less effective (L) strain; Fix^+^ (E) versus Fix^−^ (L), Fix^+^ (E) versus Fix^int^ (L), and Fix^int^ (E) versus Fix^−^ (L); that is, Fix^+^ is always effective and Fix^−^ always less effective, but Fix^int^ can be either effective or less effective, depending on the co-inoculated strain. In each case, the more effective strain was tagged with mCherry and the less effective strain with GFP. Two sets of plants, both containing six replicates of each pairwise combination, were initially set up for the collection of nodules for flow cytometry. Additional plants were set up in sets of five replicates per pairwise combination for confocal imaging of nodules at various time points.

### Harvesting

For flow cytometry, plants were harvested at 28 dpi, and for confocal microscopy they were harvested at 28, 35, and 42 dpi. Plants were harvested by removing them from the sand/vermiculite mixture and washing the roots carefully. Nodules were then imaged using a Leica M165 FC fluorescent stereo microscope and an iBright FL1500 imaging system. Nodule occupants were identified based on their fluorescence. When selecting single-occupancy nodules, the five largest of each nodule type were selected so as to account for variation due to differences in developmental stage; nodules of greatest size will be the most mature of each type, as in Westhoek *et al*. (2021). When selecting mixed nodules, all mixed nodules identifiable on the plant were selected due to the relative scarcity of mixed nodules. For single-occupancy experiments, 17 plants with five nodules of each type per plant were taken. For mixed nodule experiments, all mixed nodules were picked across seven plants.

### Confocal imaging

To assess the health of cells containing undifferentiated bacteria and bacteroids within nodules, sections were imaged using confocal microscopy. This allows the visualization of fluorescently tagged strains and the discrimination of the two strains within a mixed nodule. Nodules were placed in 8% w/v agar, and 100 μm longitudinal sections were cut through the centre of the nodule using a Leica VT1200S vibratome. Nodule slices were then imaged with a Zeiss LSM 880 Airy Scan confocal microscope and analysed with ZEN Black software. To visualize fluorescent tags, mCherry was excited using a 561 nm wavelength laser and emissions were detected between 598 nm and 649 nm, while GFP was excited using a 488 nm wavelength laser and emissions were detected between 498 nm and 562 nm.

### Flow cytometry

To identify and quantify the population sizes of undifferentiated bacteria and bacteroids within nodules, flow cytometry was used. This was applied to single-occupancy nodules from the three co-inoculant combinations (Fix^+^ versus Fix^−^, Fix^+^ versus Fix^int^, and Fix^int^ versus Fix^−^) and to mixed nodules containing Fix^+^ and Fix^−^. All nodules were prepared by placing them in a 1.5 ml Eppendorf tube with 300 μl of harvest solution (0.9% NaCl, 0.02% SILWET L-77) and crushing them with an autoclaved microcentrifuge pestle. This solution was passed through a 40 μm filter and diluted 10-fold to increase the accuracy of the flow cytometry. Flow cytometry was conducted with a Amnis® CellStream® flow cytometer (Luminex) equipped with a 488 nm and a 561 nm laser, which were used for excitation of GFP and mCherry, respectively. Flow rates were set to low speed and high sensitivity (3.66 µl min^–1^) and the flow cytometer was set to run 10 µl per sample. Analysis of flow cytometry data was carried out using the CellStream® Analysis software (Version 1.5.17). Bacterial events were defined based on custom gating parameters. Singlets and doublets were gated with a threshold of 0.4 forward scatter (FSC) aspect ratio. Bacterial singlets which emitted at 611/631 nm, when excited at 561 nm with an intensity >6000 arbitrary units (AU) were defined as red. Bacterial singlets with emission at 528/546 nm, when excited at 488 nm with an intensity >4000 AU were defined as green. Bacteroids and bacteria were defined based on size, as in Mergaert *et al*. (2006), using custom gating of the FSC detection to separate the two populations (Supplementary Fig. S1). The flow cytometer calculates a value of events per ml from the number of counts within the 10 µl as the flow cytometer assumes the sample comes from a volume of 1 ml (rather than the 300 µl we had per sample), and the sample underwent a 10-fold dilution. In order to calculate the true events per nodule, we multiplied the flow cytometer calculated value by three. Flow cytometry data are available at http://zenodo.org for the single-occupancy experiments separated by inoculum pairing and mixed nodule experiments. Citations for data are provided in the Data availability section. The dataset calculated from the flow cytometry files is also provided (Supplementary Dataset S1).

### Acetylene reduction assay

Fixation rates were measured via an acetylene reduction assay using the method described in Westhoek *et al.* (2021). Whole plants were placed into 250 ml Schott bottles with a neoprene airtight seal. Acetylene gas was added to the bottle to make up 2% of the volume. This low concentration was used to minimize the effect of acetylene on the activity of nitrogenase (Minchin *et al*., 1983). After 1 h, the amount of acetylene converted to ethylene was measured through GC.

### Statistical analysis

To test the effect of the fluorescent markers on the symbiotic characteristics of Rlv3841, we compared the fixation rates of three sets of four plants inoculated with untagged Fix^+^, Fix^+^–GFP or Fix^+^–mCherry using a Kruskal–Wallis test as the dataset did not meet the assumption of normality for a parametric test. We compared the nodulation competitiveness of the same three strains in three sets of pairwise combinations using paired *t*-tests comparing four plants for each co-inoculation.

To test for conditional sanctioning at the whole-nodule level, we subsetted the data into the three different nodule types: Fix^+^, Fix^−^, and Fix^int^, and tested whether three relevant measures of bacterial fitness and plant sanctioning behaviour were dependent on the identity of the co-inoculated strain. The chosen measures were: (i) the size of the undifferentiated bacterial population; (ii) the size of the bacteroid population; and (iii) the size of individual undifferentiated bacteria (a key measure of bacterial starvation; Shi et al., 2021). We did not measure the size of individual bacteroids because the numbers of bacteroids within some sanctioned nodules was so small as to make this measure unreliable. Both population sizes and sizes of individuals were analysed using mixed-effects models in which individual plant ID was the random effect and the identity of the co-inoculant was the fixed effect.

To test for the presence of cell-autonomous sanctioning within mixed nodules, we compared the number of bacteroids, number of bacteria, and the mean size of bacteria of the two strains within each mixed nodule using a linear mixed-effects model; the random effect was nodule ID nested within plant ID.

To get a better sense of how the plant treats mixed nodules, we compared the total number of bacteroids, total number of bacteria, and average size of bacteria in the mixed nodules with Fix^+^ and Fix^−^ single-occupancy nodules; plant ID was the random effect.

All statistical tests were carried out using R version 4.2.1 and R studio v. 2022.07.2, and graphs were produced using Graph Pad version 9. An R markdown document is provided (Supplementary Appendix S1). For simplicity, the numbers of bacteria and bacteroids are always presented log_10_ transformed within figures. This log transformation was only required for a subset of analyses in order to meet the assumptions of equal variance and normality. These were assessed by visual inspection of residual plots (see R markdown file, Supplementary Appendix S1). Where log10 transformation was carried out for analysis, the statistical outputs have been back-transformed for readability. Back transformation was carried out using the formulae given in Supplementary Fig. S2.

When testing the assumption of normality for statistical tests where the sample size was <50, the Shapiro–Wilks test was used. When the sample size was >50, the visual inspection of Q–Q plots was used instead of the Shapiro–Wilks test as recommended in the literature (Mishra *et al*., 2019). Where Shapiro–Wilks has been used the values are reported. Q–Q plots are provided in Supplementary Appendix S1.

## Results

### Fluorescently tagging strains does not affect symbiotic characteristics of Rlv3841

We first tested that fluorescent marking does not alter the competitiveness of rhizobial strains. There was no significant difference in the number of nodules occupied by each strain on plants inoculated with equal numbers of untagged Rlv3841 versus its mCherry- and GFP-tagged versions (Fix^+^ versus Fix^+^–mCherry: estimate=22.750, SE=13.288, *t*=1.712, *P*>0.05, df=6; Fix^+^ versus Fix^+^–GFP: estimate= −5.75, SE=20.84, *t*= −0.276, *P*>0.05, df=6). Similarly, plants co-inoculated with mCherry- and GFP-tagged Rlv3841 did not have significant differences in nodules occupied by each strain (Fix^+^–GFP versus Fix^+^–mCherry: estimate= −12.250, SE=11.265, *t* = −1.087, *P*>0.05, df=6) For all three datasets, the Shapiro–Wilks test *P*-value exceeded 0.05. In addition, peas inoculated singly with the three strains did not differ significantly in fixation rates per nodule (Shapiro–Wilks: *P*=0.0316. Kruskal–Wallis: df=2, *P*>0.05).

### Conditional sanctioning reduces the number of bacteroids and the size of bacteria

In Westhoek *et al.* (2021), it was shown that at 28 dpi the number of viable bacteria in sanctioned nodules had not decreased compared with unsanctioned nodules despite the significantly smaller size of sanctioned nodules. Therefore, we hypothesized that within sanctioned nodules the number of non-replicating bacteroids must be significantly lower than in unsanctioned nodules. At the same time, the lower carbon supply to these sanctioned nodules would cause a reduction in the size of the undifferentiated bacteria due to starvation (Shi *et al*., 2021). Therefore, the number of bacteria and bacteroids and the size of bacteria within nodules were measured through flow cytometry.

**Fig. 1.**
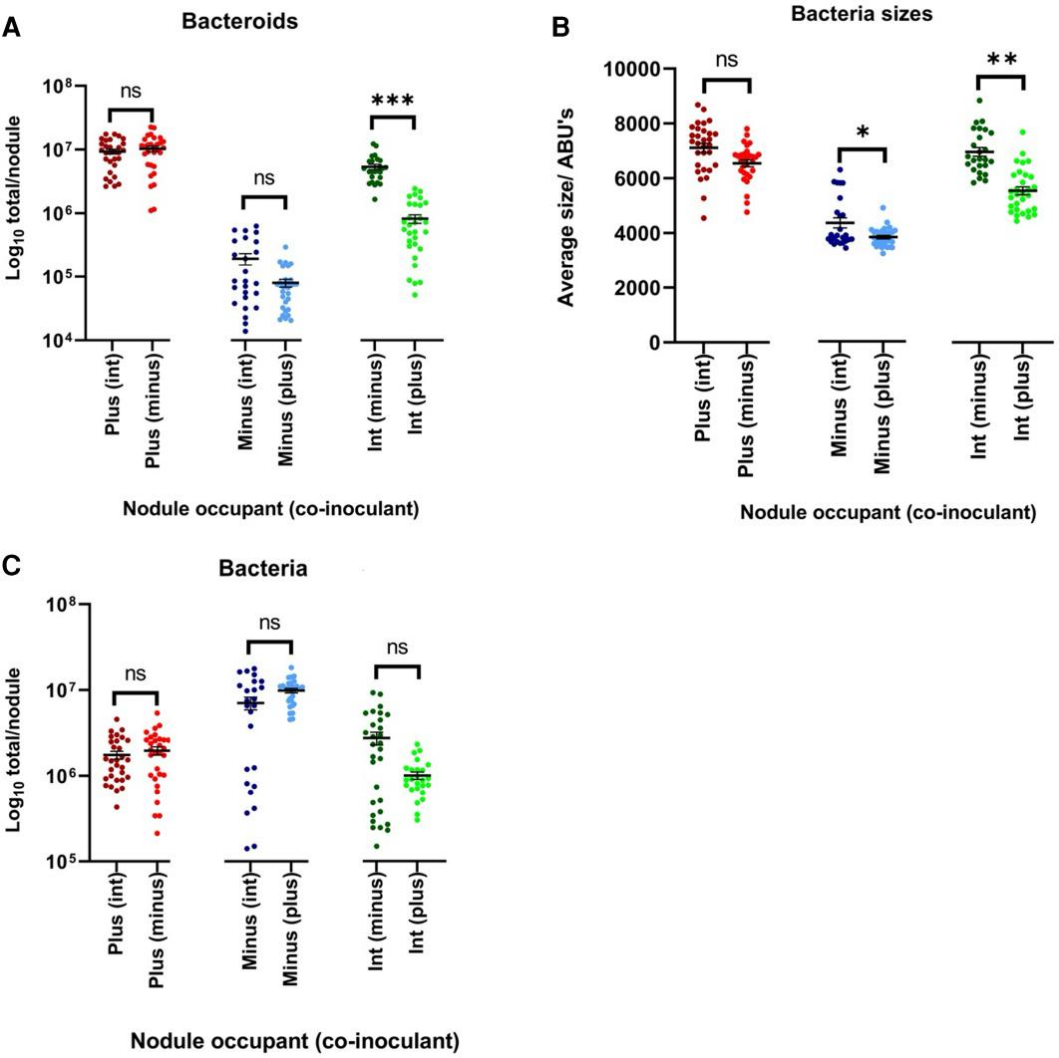
After 28 d, plant-imposed sanctions reduce the number of bacteroids and bacterial size, but not the number of bacteria. The number of bacteroids (A), the size of bacteria (B), and the number of bacteria (C), measured by flow cytometry, in nodules from pea plants co-inoculated with two strains of rhizobia. Data are split into different single-strain nodule types (Fix^+^, Fix^int^, or Fix^−^). The identity of the co-inoculant is given in parentheses. Horizontal bars give the mean value with 1 SE. Significance level from paired *t*-test ***<0.001; **<0.01; *<0.05; ns, not significant. All nodules were harvested at 28 d post-inoculation. Strains were tagged with a fluorescent protein. Fix^+^ was always tagged with mCherry, Fix^−^ was always tagged with GFP, and Fix^int^ was tagged with mCherry when co-inoculated with Fix^−^ and with GFP when co-inoculated with Fix^+^.

Nodules occupied by the intermediate-fixing strain contained significantly fewer bacteroids when co-inoculated with Fix^+^ rather than Fix^−^ bacteria. Thus conditionally sanctioned nodules have reduced bacteroid numbers. As expected, the number of bacteroids in Fix^+^ and Fix^−^ nodules did not significantly vary depending on the co-inoculated strain (Fig. 1A) (Table 2). This is because, in our experiments, Fix^+^ nodules always contain the most effective strain and therefore will not be sanctioned, while Fix^−^ nodules always contain the less effective strain and so therefore are sanctioned.

**Table 2.** Impact on number of bacteroids of the focal strain with different co-inoculants

Strain (co-inoculant comparisons)	Difference in number of bacteroids	SE	*t*	*P*	*n*
Fix^+^ (Fix^int^ and Fix^−^)	970 769	2 039 541	0.476	>0.05	60
Fix^int^ (Fix^+^ and Fix^−^)	−4.29×10^6^ (log_10_)	2.02×10^5^ (log_10_)	6.818	7.74×10^−5^	55
Fix^−^ (Fix^+^ and Fix^int^)	44 647(log_10_)	21 731(log_10_)	−1.806	>0.05	55

Mixed-effects models were used to compare the number of bacteroids within a nodule when co-inoculated with different strains. Data that have been log_10_ transformed are indicated and presented back transformed. *P*-values exceeding 0.05 are reported as >0.05. The value for difference in bacteroid number is given for the more versus less effective co-inoculant. The *t*- and *P*-value are taken from the mixed-effects model, and *n* is the number of nodules.

Undifferentiated bacteria from Fix^int^ nodules were significantly smaller when co-inoculated with a Fix^+^ rather than with a Fix^−^ strain. Thus, conditional sanctioning reduces the size of undifferentiated bacteria within nodules. As expected, undifferentiated bacteria from Fix^+^ nodules were not significantly altered in size when co-inoculated with Fix^int^ or Fix^−^ strains. Surprisingly, undifferentiated bacteria from Fix^−^ nodules were significantly smaller when co-inoculated with a Fix^+^ strain, but not when co-inoculated with a Fix^int^ strain (Fig. 1B) (Table 3).

**Table 3.** Impact on size of undifferentiated focal bacterial size of strains with different co-inoculants

Strain (co-inoculant comparisons)	Difference in size of bacteria	SE	*t*	*P*	*n*
Fix^+^ (Fix^int^ and Fix^−^)	561.3	301.6	1.861	0.0923	60
Fix^int^ (Fix^+^ and Fix^−^)	−1417.6	405.9	−3.492	0.0068	55
Fix^−^ (Fix^+^ and Fix^int^)	−450 (log_10_)	170 (log_10_)	−2.566	0.03	55

Mixed-effects models were used to compare the number of bacteroids within a nodule when co-inoculated with different strains. Data that have been log_10_ transformed are indicated and presented back transformed. *P*-values exceeding 0.05 are reported as >0.05. The value for difference in bacteroid number is given for the more versus less effective co-inoculant. The *t*- and *P*-value are taken from the mixed-effects model, and *n* is the number of nodules.

Finally, the number of undifferentiated bacteria within a nodule did not depend on the identity of the co-inoculant for any of our nodule types (Fix^+^ nodules, Fix^−^ nodules, or Fix^int^ nodules). This is consistent with previous studies which found no significant change in colony-forming units (CFU) due to conditional sanctioning at 28 dpi [although it does change at 56 dpi (Westhoek *et al*. (2021)]. Overall, conditional sanctioning therefore reduces both bacteroid number and the size of undifferentiated bacteria. However, co-inoculant does not alter the number of bacteria at 28 dpi (Fig. 1C; Table 4).

**Table 4.** Impact on number of undifferentiated bacteria of each strain with different co-inoculants

Strain (co-inoculant comparisons)	Difference in number of bacteria	SE	*t*	*P*	*n*
Fix^+^ (Fix^int^ and Fix^−^)	−212 437	472 483	−0.450	>0.05	60
Fix^int^ (Fix^+^ and Fix^−^)	−5.81×10^5^ (log_10_)	1.17×10^5^ (log_10_)	0.855	>0.05	55
Fix^−^ (Fix^+^ and Fix^int^)	2.79×10^6^	2.01×10^6^	1.391	>0.05	55

Mixed-effects models were used to compare the number of bacteroids within a nodule when co-inoculated with different strains. Data that have been log_10_ transformed are indicated and presented back transformed. *P*-values exceeding 0.05 are reported as >0.05. The value for difference in bacteroid number is given for the more versus less effective co-inoculant. The *t*- and *P*-value are taken from the mixed-effects model, and *n* is the number of nodules.

### Conditional sanctioning changes nodule cell morphology

**Fig. 2.**
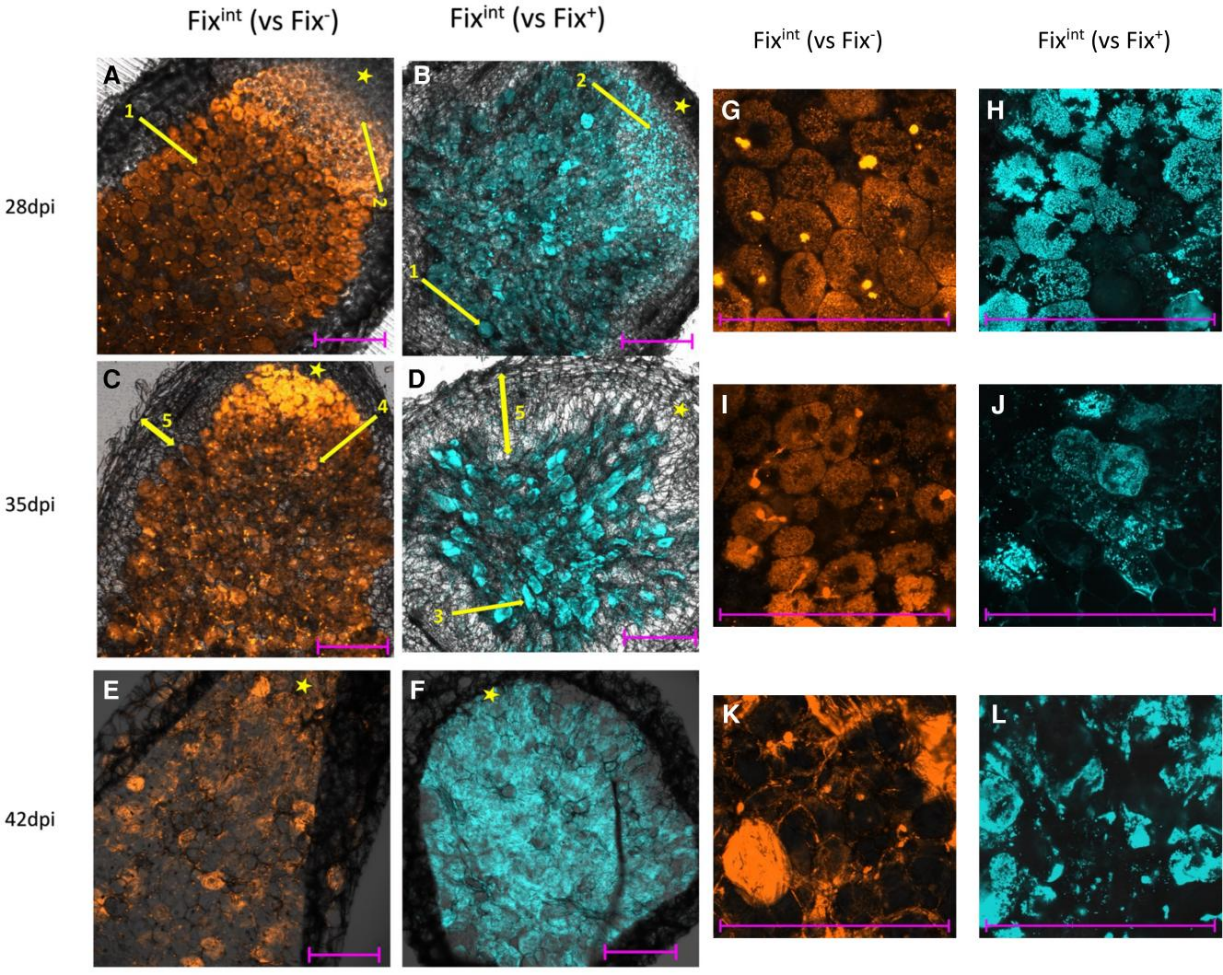
Fix^int^ nodules co-inoculated with the Fix^+^ strain change cell morphology earlier than those co-inoculated with the Fix^−^ strain. Confocal microscopy images of pea nodules containing either: a Fix^int^ strain (A), (C), and (E) tagged with mCherry (orange), on pea plants co-inoculated with Fix^−^, or a Fix^int^ strain (B), (D), and (F) tagged with GFP (blue), on pea plants co-inoculated with Fix^+^. Nodules were harvested at 28, 35, and 42 days post-inoculation (dpi). Longitudinal nodule slices (100 µm n thickness) were imaged. The tip of the nodule is indicated by a star. At 28 dpi, regardless of co-inoculant, infected cells were spherical (1) and an actively dividing meristem is visible (2). At 35 dpi, the Fix^int^ nodules co-inoculated with Fix^+^ were no longer spherical (3), in contrast to those seen in the Fix^int^ nodules co-incoulated with Fix^−^ (4). In addition, the area of infected cells in sanctioned nodules shows clear withdrawal from the edge of the nodule compared with unsanctioned nodules (5). At 42 dpi, there were very few remaining spherical cells in either sanctioned or unsanctioned nodules. At each time point, additional images taken at 4× higher magnification are provided for each nodule type (G–L) Scale bar=200 µm.

After 28 dpi, the size of sanctioned whole nodules is clearly reduced, as seen in Westhoek *et al*. (2017). However, to study the effects on cellular morphology of sanctioned nodules, plants were co-inoculated with all three combinations of bacterial strains (Fix^+^ versus Fix^−^, Fix^+^ versus Fix^int^, Fix^int^ versus Fix^−^) and nodules were imaged at 28, 35, and 42 dpi (Supplementary Fig. S3). At 28 dpi, all sections taken from nodules containing the Fix^int^ strain looked similar, regardless of whether the co-inoculated strain was Fix^−^ (Fig. 2A) or Fix^+^ (Fig. 2B). However, at 35 dpi, nodules containing the Fix^int^ strain were visibly affected when the co-inoculated strain was Fix^+^. Infected cells within Fix^int^ nodules were irregularly shaped and the infected region had retracted from the nodule edge (Fig. 2D). By comparison, when the co-inoculated strain was Fix^−^, most cells retained a round morphology, and were not visibly different from Fix^int^ nodules at 28 dpi (compare Fig. 2C with Fig. 2A and B). At 42 dpi, all Fix^int^ nodules showed clear changes to cell morphology, which were most pronounced when co-inoculated with Fix^+^ (Fig. 2F), rather than Fix^−^ (Fig. 2E). Therefore, conditional sanctioning induces a premature change to the cellular morphology of nodules.

### Conditional sanctioning is not cell-autonomous in pea

Cell-autonomous sanctioning predicts that in nodules containing more than one strain (mixed nodules) the plant will differentiate between the strains and sanction cells containing the less effective strain. This would result in fewer bacteroids and smaller undifferentiated bacteria of the less effective relative to the more effective strain. We tested this theory in Fix^+^ and Fix^−^ mixed nodules, as this is the most extreme difference in fixation rates between strains available, by measuring the number of bacteroids and bacteria and the size of bacteria through flow cytometry.

A prediction for the effect on numbers of bacteria could not be made as within the single-occupancy nodule there was not a consistent effect of conditional sanctioning on the number of bacteria compared with unsanctioned nodules. However, there was a clear increase in the number of bacteria within Fix^−^ nodules, as had been observed in Westhoek *et al*. (2021).

**Fig. 3.**
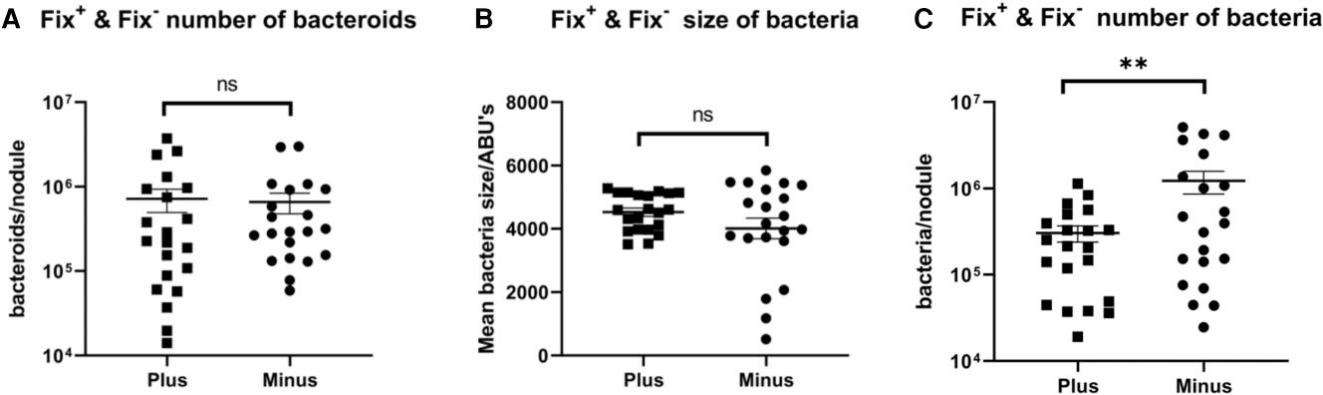
The two strains within a mixed nodule are not sanctioned independently. The number of bacteroids (A), the size of bacteria (B), and the number of bacteria (C) (measured by flow cytometry) for the two strains within mixed nodules on pea plants. Mixed nodules contained both a Fix^+^ and a Fix^−^ strain. All nodules were harvested 28 d post-inoculation. Horizontal bars give the mean value with 1 SE. Regardless of strain identity, there was no-significant difference in the number of bacteroids or the size of bacteria within a mixed nodule. However, there were significantly more Fix^−^ bacteria than Fix^+^ bacteria. Significance level from paired *t*-test: **<0.01; ns, not significant. Strains were tagged with a fluorescent protein. Fix^+^ was tagged with mCherry, Fix^−^ was tagged with GFP.

When comparing the number of bacteroids of each of the two strains within mixed nodules there was no significant difference between the number of Fix^+^ and Fix^−^ (log_10_ transformed: difference in number of bacteroids=1.133×10^5^, SE=73528, *t*=1.455, *P*>0.05, *n*=21) (Fig. 3A). If peas were able to specifically sanction cells containing ineffective strains within a mixed nodule, the numbers of ineffective bacteriods would be expected to be reduced. Therefore, the data are consistent with peas being unable to differentiate between the two strains within a mixed nodule.

There was no significant difference in the size of bacteria within mixed nodules between the more effective and the less effective strain (difference in size=511, SE=267, *t*=1.916, *P*>0.05, *n*=21) (Fig. 3B). This is also consistent with an absence of cell-autonomous sanctioning, as sanctions had a clear impact on the size of undifferentiated bacteria in single-occupancy nodules (Fig. 1B) that is not present in mixed nodules.

Intriguiginly, there were significantly fewer Fix^+^ bacteria compared with the number of Fix^−^ bacteria within mixed nodules (log_10_ transformed: difference in number of bacteria=−1.89×10^5^, SE=48338, *t*= −3.675, *P*=0.0015). While the mechanism for this is unclear, it suggests the possibility that bacterial cell divison or infection thread ramification is affected by bacteroid formation and perhaps nitrogen fixation.

For all three datasets (number of bacteroids, number of bacteria, and size of bacteria) the Shapiro–Wilks test *P*-value exceeded 0.05.

Based on these results, mixed nodules of pea do not show cell-autonomous sanctioning. Since this makes piggybacking in mixed nodules a potential route to success for a strain less effective at fixing N_2_, we also examined how mixed nodules were treated at the whole-nodule level by the plant.

### Mixed nodules are sanctioned

If mixed nodules are sanctioned at the whole-nodule level, then mixed nodules should be sanctioned in the same manner as a single-strain intermediate fixing nodule. This is because, theoretically, the total amount of nitrogen fixed by a mixed nodule will be the average of the two strains. As such, the fixation output of a mixed nodule should be intermediate to the two strains on their own. As seen in Fig. 1, when a Fix^int^ nodule was sanctioned, it had a significantly reduced number of bacteroids and smaller bacteria. To test this, the total number of bacteroids, bacterial size, and bacterial numbers in mixed nodules were compared with the two single-occupancy nodules taken from plants co-inoculated with Fix^+^ and Fix^−^. Intermediate sanctioning of mixed nodules predicts that mixed nodules will contain more bacteroids and larger bacteria than the less effective Fix^−^ nodule while having fewer bacteroids and smaller bacteria than the more effective single Fix^+^ nodule, as was seen for sanctioned Fix^int^ nodules (see Fig. 1).

**Fig. 4.**
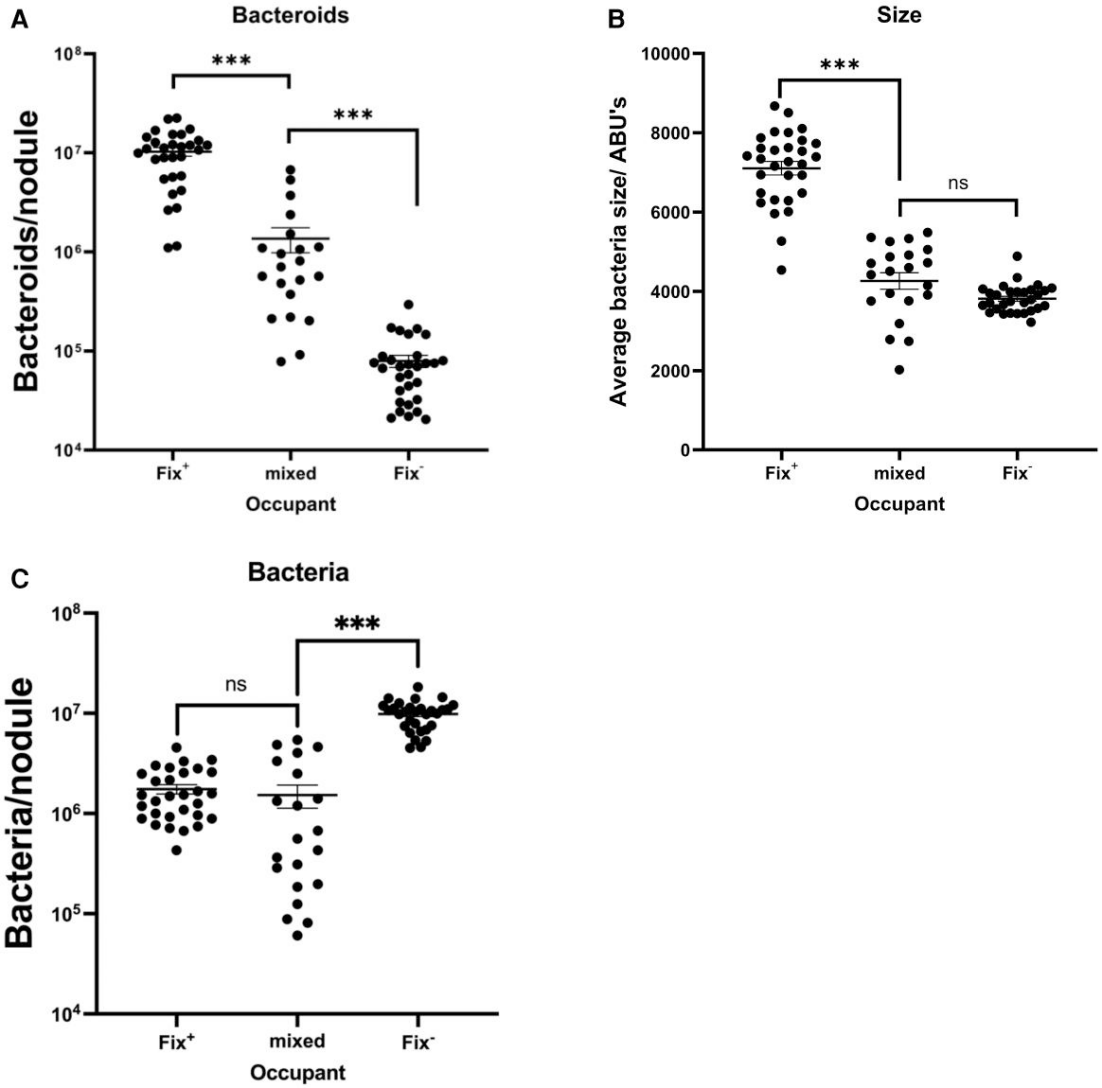
Mixed nodules are sanctioned, but less severely than single-strain nodules containing the less effective strain. The number of bacteroids (A), the size of undifferentiated bacteria (B), and the number of undifferentiated bacteria (C) within pea nodules measured by flow cytometry. Nodules contained either a Fix^+^ strain, a Fix^−^ strain, or were mixed nodules containing Fix^+^ and Fix^−^. The mixed nodules are compared with the single-occupancy Fix^+^ and Fix^−^ nodules. Mixed nodules contained significantly fewer bacteroids and significantly smaller bacteria, but did not show a significant difference in the number of bacteria when compared with the Fix^+^ nodules. Mixed nodules contained significantly more bacteroids and significantly fewer bacteria than Fix^−^ nodules but did not significantly differ in the size of the bacteria. Bars indicate the mean value and error bars are 1 SE. All nodules were harvested 28 d post-inoculation. Significance levels from *t*-test: ***<0.001; ns, not significant. Strains were tagged with a fluorescent protein. Fix^+^ was tagged with mCherry, Fix^−^ was tagged with GFP.

As predicted, the number of bacteroids within a mixed nodule was significantly lower than the number within the Fix^+^ only nodule (log_10_ transformed: difference in number of bacteroids= −7.17×10^6^, SE=3.02×10^6^, *t*=7.232, *P*<0.001) (Fig. 4A). The mixed nodules also contained significantly more bacteroids than the Fix^−^ only nodules (log_10_ transformed: difference in number of bacteroids=7.08×10^5^, SE=22 737, *t*=7.914, *P*<0.001) (Fig. 4A). These results are consistent with mixed nodules being treated at the whole-nodule level and sanctioned as an intermediate fixing strain.

As predicted, bacteria within mixed nodules were significantly smaller than those within the more effective Fix^+^ nodules (difference in size= −2814, SE=296, *t*=9.513, *P*<0.001) (Fig. 4B) This dramatic decrease in size is consistent with mixed nodules being intermediately sanctioned. However, there was no significant difference in size compared with the less effective nodule control (difference in size=475, SE=296, *t*=1.608, *P*>0.05) (Fig. 4B). This suggests that the change in size of bacteria is only dramatic when comparing a sanctioned nodule relative to the unsanctioned Fix^+^ nodule. In contrast, there is not a dramatic change in size when comparing two different types of sanctioned nodules such as a Fix^−^ nodule or a mixed nodule of Fix^+^ and Fix^−^.

There was no significant difference in the number of undifferentiated bacteria within the mixed nodules when compared with the number within the more effective Fix^+^ nodules (log_10_ transformed: difference in number of bacteria=3.63×10^5^, SE=5.83×10^5^, *t*=0.918, *P*>0.05). In contrast, there were significantly fewer bacteria within the mixed nodule when compared with the less effective Fix^−^ nodule (log_10_ transformed: difference in number of bacteria= −6.54×10^6^, SE= −3.65×10^6^, *t*=7.914, *P*<0.001).

### Changes to cell morphology are not cell-autonomous in pea

**Fig. 5.**
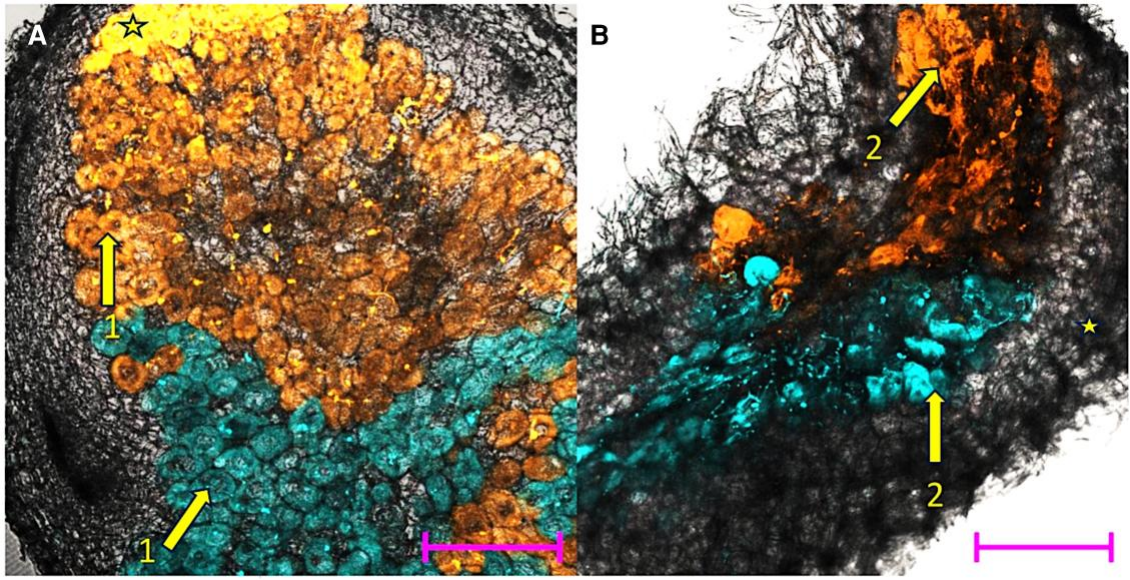
The two strains within a mixed nodule do not senesce at different times despite varying in relative effectiveness. Confocal microscopy images of pea plant nodules containing multiple strains. Images were taken 35 d post-inoculation. The two strains within the nodule were a Fix^+^ (orange: mCherry tagged) and Fix^−^ (blue: GFP tagged) (A and B) strain. These nodules showed either no signs of sanctioning (A) or clear signs of sanctioning (B). When unsanctioned (A), all cells of both strains remain spherical and intact (1). When sanctioned (B), cells of both strains burst open (2). Longitudinal nodule slices (100 µm in thickness) were taken. Yellow stars indicate the tip of the nodule. Scale bar=200 µm.

Within mixed nodules there was no evidence of any differences in cell morphology between cells occupied by the Fix^+^ or Fix^−^ strain. If the nodule was unsanctioned (Fig. 5A), then all of the cells within the nodule were healthy, regardless of the strain occupying the cell. In contrast, if a nodule was sanctioned (Fig. 5B), then—as for a single-occupancy nodule (Fig. 2D)—infected cells within the nodule lost their typical spherical morphology and some burst open. However, this was equally likely to affect cells containing the more or the less effective strain. Therefore, we have seen no evidence to support cell-autonomous sanctioning.

## Discussion

We show that after 28 d the size of undifferentiated bacteria (Fig. 1B) and the number of bacteroids (Fig. 1A), but not the number of undifferentiated bacteria (Fig. 1C), significantly decreased within a sanctioned nodule. Peas sanctioning nodules containing less effective strains after 28 d agrees with previous studies (West *et al*., 2002; Kiers *et al*., 2003; Oono *et al*., 2011; Westhoek *et al*., 2017). Consistent with the findings of Westhoek *et al*. (2021), the number of undifferentiated bacteria was not significantly lower in sanctioned nodules at 28 dpi. It may be theorized that the lack of difference (and, in the case of Fix^−^, a large increase) in numbers of bacteria within sanctioned nodules is as a result of saprophytic activity by the bacteria within the sanctioned nodule as bacteroids die from carbon starvation (Timmers *et al*., 2000). Our data are also consistent with conditional sanctioning, as the fate of nodules, the number of bacteroids, and nodule internal morphology infected by a Fix^int^ strain depend on whether the co-inoculated strain is Fix^+^ or Fix^−^ (Westhoek *et al*., 2021). In this study, we also used flow cytometry to quantify and analyse the bacteroid and bacterial populations. This revealed that the decrease in size of sanctioned nodules at 28 dpi is linked to a significant drop in the bacteroid population.

Intriguingly, the number of bacteroids within the sanctioned Fix^int^ nodules was still clearly higher than that within the Fix^−^ nodules (Fig. 1A). This appears to show that Fix^int^ nodules are not as severely sanctioned as Fix^−^ nodules. This might be explained by the fact that a Fix^−^ nodule, which never fixes nitrogen, will be sanctioned at the earliest possible time point. In contrast, an intermediately fixing nodule will initially be supported by the plant before eventually being sanctioned once it is clear that the output of the nodule is lower than that of other nodules. Therefore, these intermediate nodules will reach a later developmental stage before the onset of sanctioning, resulting in more bacteroids.

Within sanctioned nodules, there was also a reduction in the size of undifferentiated bacteria. The importance of bacterial size to the measurement of sanctions is that a reduction in size has previously been shown as a direct consequence of starvation (Shi *et al*., 2021). Given that in Westhoek *et al*. (2021) it was shown that sanctioned nodules experience a significant drop in carbon supply, it would be expected that bacteria would starve within these nodules, leading to a drop in size.

While bacterial numbers remain high, and were much higher in non-fixing nodules (Fig. 1C), the bacteria were much smaller (Fig. 1B), pre-dating the collapse in their population at 56 dpi (Westhoek *et al*., 2021). Undifferentiated intermediate-fixing bacteria were also significantly smaller when co-inoculated with a Fix^+^ strain. While the size of undifferentiated Fix^−^ bacteria changed with the identity of the co-inoculant, the estimated size difference was smaller, and close to the significance threshold.

Compared with unsanctioned nodules, sanctioned nodules have an altered internal structure (Fig. 2). Previous studies have linked these morphological changes to nodule senescence (Regus *et al*., 2017). By tracking changes through nodule development, we measured the sequence of events that take place throughout sanctioning. Sanctioned nodules are smaller and more spherical by 16 dpi (Westhoek *et al*., 2017). By 28 dpi, there was also a drop in the number of bacteroids and the size of undifferentiated bacteria (Fig. 1); however, there was no visible change in the internal structure of sanctioned nodules before 35 dpi (Fig. 2). All of these changes appear to precipitate the collapse in the undifferentiated bacteria population which is observed by 56 dpi (Westhoek *et al*., 2021).

It has been proposed that cell-autonomous sanctions occur within mixed nodules (Daubech *et al*., 2017; Regus *et al*., 2017). Cell-autonomous sanctioning predicts that plant cells containing a less effective bacterial strain will be sanctioned, while plant cells within the same nodule containing a more effective strain will not. If true, then the less effective strain within a mixed nodule should have significantly fewer bacteroids and the size of bacteria should be significantly smaller. Furthermore, we would expect to see premature senescence of plant cells containing the less effective bacterial strain compared with the more effective strain.

Our results did not show any of the above predicted effects within mixed nodules in pea. First, the number of bacteroids was not significantly different (Fig. 3A). Second, the size of bacteria within mixed nodules was not significantly different (Fig. 3B). Finally, plant cells within mixed nodules underwent senescence simultaneously, regardless of effectiveness (Fig. 5). Therefore, in peas, there is no evidence for cell-autonomous sanctions on the less effective strain within a mixed nodule.

It was also shown that within mixed nodules, the number of undifferentiated bacteria of the Fix^−^ strain was significantly higher than that of the Fix^+^ strain (Fig. 3C). Furthermore, the number of undifferentiated bacteria is also higher in single-occupancy Fix^−^ relative to Fix^+^ nodules (Fig. 1C). As considered above, the mechanism for this is unclear but suggests that bacterial cell divison in infection threads or thread ramification is increased when the bacterial strain is Fix^−^. This suggests some sort of feedback from the process of bacteroid differentiation and subsequent nitrogen fixation. For example, once an infected cell starts fixing nitrogen, it might feed-back inhibit bacterial replication or infection thread ramification. With regard to this possibility, it is notable that this increase in bacterial number does not occur in Fix^int^ nodules even when sanctioned by Fix^+^ nodules. This would make sense because initially Fix^int^ nodules do fix nitrogen and are only subsequently sanctioned by the presence of Fix^+^ nodules.

However, while we have shown that these mixed nodules are not sanctioned cell-autonomously, they do appear to be treated in a different manner to the wholly effective or less effective nodules. Whole mixed nodules are sanctioned at a level intermediate to the two strains comprising them in isolation (Fig. 4). This is to be expected as a mixed nodule will output nitrogen at a level somewhere between the nodules of two strains in isolation. Therefore, in the same way that Fix^int^ nodules had more bacteroids than Fix^−^ nodules, we may conclude that mixed nodules remain unsanctioned for longer than a Fix^−^ nodule due to the output of some nitrogen, but as with the Fix^int^ nodules they are eventually sanctioned as they do not output to the same level as the Fix^+^ nodule. The intermediate level sanctioning of mixed nodules demonstrates that plants can distinguish between nodules of subtly different fixation effectiveness. This supports our conclusion that sanctioning must be controlled through an extraordinarily sensitive response to the overall nitrogen status of a plant.

It should be noted that, while the mixed nodules contained smaller bacteria than the Fix^+^ nodules, they did not contain larger bacteria than the Fix^−^ nodules as we would have predicted (Fig. 4B). It is possible therefore that the effect of sanctioning on the size of bacteria is not as sensitive as the effect on bacteroid numbers.

When comparing the number of bacteria within mixed nodules with the single-occupancy nodules, the mixed nodules contained significantly fewer bacteria than the Fix^−^ nodules and showed no significant difference from the Fix^+^ nodules (Fig. 4C). This result suggests that while, as previously proposed, there may be some feedback between bacteroid differentiation and fixation, it may be that it is only in the extreme case of a completely non-fixing nodule that the conditions are correct for the amplification of bacterial numbers seen in the Fix^−^ nodules.

The lack of evidence for cell-autonomous sanctions aligns with Agtuca *et al*. (2020), who demonstrated that the metabolic profile of sectors of mixed nodules containing different strains did not show significant differences. However, our results contrast with studies which have found evidence for cell-autonomous sanctions based on variation in the timing of senescence, such as Regus *et al*. (2017) and Daubech *et al*. (2017). The differences may be explained by variation in the legume–rhizobium system used. For example, Daubech *et al*. (2017) used the *Mimosa pudica*—*Cupriavidus taiwanensis* symbiosis, while Regus *et al*. (2017) used both the *Acmispon strigosus—Bradyrhizobium* and the *Lotus japonicus—Mesorhizobium* symbioses. Finally, Agtuca *et al*. (2020) used the *Glycine max*—*Bradyrhizobium japonicum* symbiosis.

The studies to date have, therefore, focused entirely on non-terminally differentiating bacteroids [the *M. pudica*—*C. taiwanensis* symbiosis being an example of an indeterminate nodule containing non-terminally differentiated bacteroids (Marchetti *et al*., 2011)]. This is important for two reasons. Firstly, it emphasizes the importance of this study as having studied mixed-nodule sanctioning in a system with terminally differentiated bacteroids. Secondly, in Denison (2000), it was hypothesized that cell-autonomous sanctioning is not feasible as a strategy to combat piggybacking in systems such as pea, as these sanctions would impact the non-reproductive bacteroids and not the undifferentiated bacteria. However, no such problem would exist in systems with non-terminally differentiated bacteroids. Therefore, the absence of cell-autonomous sanctions in the *G. max*—*B. japonicum* symbiosis in the study of Agtuca *et al*. (2020) would imply that, in at least some cases, cell-autonomous sanctions have not emerged for other reasons. A comprehensive understanding of sanctions, particularly cell-autonomous sanctions, will therefore require the continued use of multiple experimental systems.

We have shown that the sanctioning of less effective nodules within the pea–rhizobia symbiosis is conditional in nature and occurs at the nodule level. In Fix^+^/Fix^int^-co-infected plants, nodules containing Fix^int^ are sanctioned while in Fix^int^/Fix^−^-co-infected plants Fix^int^ nodules are not sanctioned. This suggests that conditional sanctioning requires comparison between nodule-specific and global nitrogen signals. This might be achieved through the interaction between a nitrate receptor such as the Nitrate transreceptor NRT1.1, which transports nitrate as well as detecting nitrate levels (Zhang *et al*., 2019) and the NIN-like Proteins (NLPs) 1 and 4, which are essential for the nitrate-based regulation of nodule maturation (Lin *et al*., 2021). In high nitrate conditions, NLP1/4 inhibit cytokinin biosynthesis. Cytokinin biosynthesis drives nodule maturation through the activation of a signal cascade through the Cytokinin-responsive element CRE1 (Lin *et al*., 2021), which acts to promote expression of the *cep* and *cle* genes (Laffont *et al*., 2020). However, how the nitrogen output of individual nodules is detected by and compared with the global nitrogen status remains unclear. The mechanism of sanctions on individual nodules may be achieved through nodule-specific proteins responding to nitrogen levels. One possibility is SnRK1 which, when phosphorylated by the DMI2 kinase in response to Nod factor, phosphorylates malate dehydrogenase 1 and 2, leading to increased malate production and supply to bacteroids (Guo *et al*., 2023). It is therefore a prime candidate for how legumes would reward nodules containing effective strains, as well as to punish nodules containing less effective strains. However, these remain speculations about signalling pathways whose elucidation may aid future efforts to engineer symbioses and in the selection of more effective nitrogen-fixing bacteria.

We now have clear evidence that the change in nodule morphology shown by Westhoek *et al*. (2017) is driven by a reduction in the number of bacteroids. This precedes the changes to the internal structure of sanctioned nodules. The number of undifferentiated bacteria drops at a later time point. We have also shown that, while the plant is unable to discriminate between effective and less effective strains within a mixed nodule, ‘piggybacking’ on an effective strain does not provide an obvious route by which to evade sanctions. We propose that the potential benefits are limited, because peas appear to view mixed nodules with a non-trivial proportion of the piggybacking strain as less effective, and sanctions them accordingly.

## Supplementary Material

erag191_Supplementary_Data

## Data Availability

Flow cytometry data are available at zenodo.org with separate datasets for each single-occupancy inoculum pairing and one dataset for mixed nodule data: Underwood (2025a) P. sativum nodules, Fix Plus and Fix Minus nodules [Dataset]. Zenodo. https://doi.org/10.5281/zenodo.14989546; Underwood (2025b) P. sativum Fix Plus and Fix Int nodules [Dataset]. Zenodo. https://doi.org/10.5281/zenodo.14989639; Underwood (2025c) P. sativum Fix Int and Fix Minus nodules [Dataset]. Zenodo. https://doi.org/10.5281/zenodo.14989691; and Underwood (2025d) P. sativum Mixed nodules [Dataset]. Zenodo. https://doi.org/10.5281/zenodo.14989800.

## Supplementary data

The following supplementary data are available at *JXB* online.

Fig. S1. Image of flow cytometry output showing bacteroid and undifferentiated bacterial populations differentiated by size based on forward scatter.

Fig. S2. Formulae for back-transformation for outputs of analyses of log_10_-transformed data.

Fig. S3. Representative confocal images of nodule sections of both nodule types from all three co-inoculation combinations (Fix^+^ versus Fix^−^, Fix^+^ versus Fix^int^, Fix^int^ versus Fix^−^) at 28, 35, and 42 d post-inoculation.

Appendix S1. R markdown file of statistical analysis.

Dataset S1. Flow cytometry data, analysed using custom gating to calculate the number of undifferentiated bacteria and bacteroids in single-occupancy and mixed nodules, and acetylene reduction assay and nodulation results from fluorescent tag control experiments.
