# Supplementary material for: Pea plants conditionally sanction less effectively fixing rhizobia at the level of whole nodules rather than single cells
Source: J Exp Bot. 2026 Apr 27;77(14):4761–72. doi: 10.1093/jxb/erag191 (PMC13415959; doi:10.1093/jxb/erag191)

The following supplementary figures are available for this article:

Fig. S1 Image of flow cytometry output showing bacteroid and undifferentiated bacterial populations differentiated by size based on Forward Scatter (FSC)

Fig. S2 Formulae for back transformation for outputs of analyses of log_{10} transformed data

Fig. S3 Representative confocal images of nodule sections of both nodule types from all three co-inoculation combinations (Fix^+ vs Fix^-, Fix^+ vs Fix^{int}, Fix^{int} vs Fix^-) at 28-, 35- and 42-days post inoculation.

Fig. S1 Nodules were picked 28 days post inoculation, crushed, and passed through a flow cytometer. The events were gated based on size to separate out bacteria and bacteroid events (A). There are two clear populations of different within a nodule. The larger population are the bacteroids. The gating line is shown in red at approximately 8000 arbitrary units FSC. Events were also gated based on aspect ratio to identify singlets and doubles (B). Gating line is at 0.400 FSC aspect ratio. To identify bacteria gating of fluorescence was used as bacteria were fluorescently tagged with mCherry or GFP. GFP bacteria were gated at values above 4000 arbitrary units (C). Nodules containing mCherry tagged bacteria showed negligible values for GFP fluorescent events (D). Bacteria tagged with mCherry were gated at values above 6000 arbitrary units (E). Nodules containing GFP tagged bacteria showed negligible values for GFP fluorescent events (F).

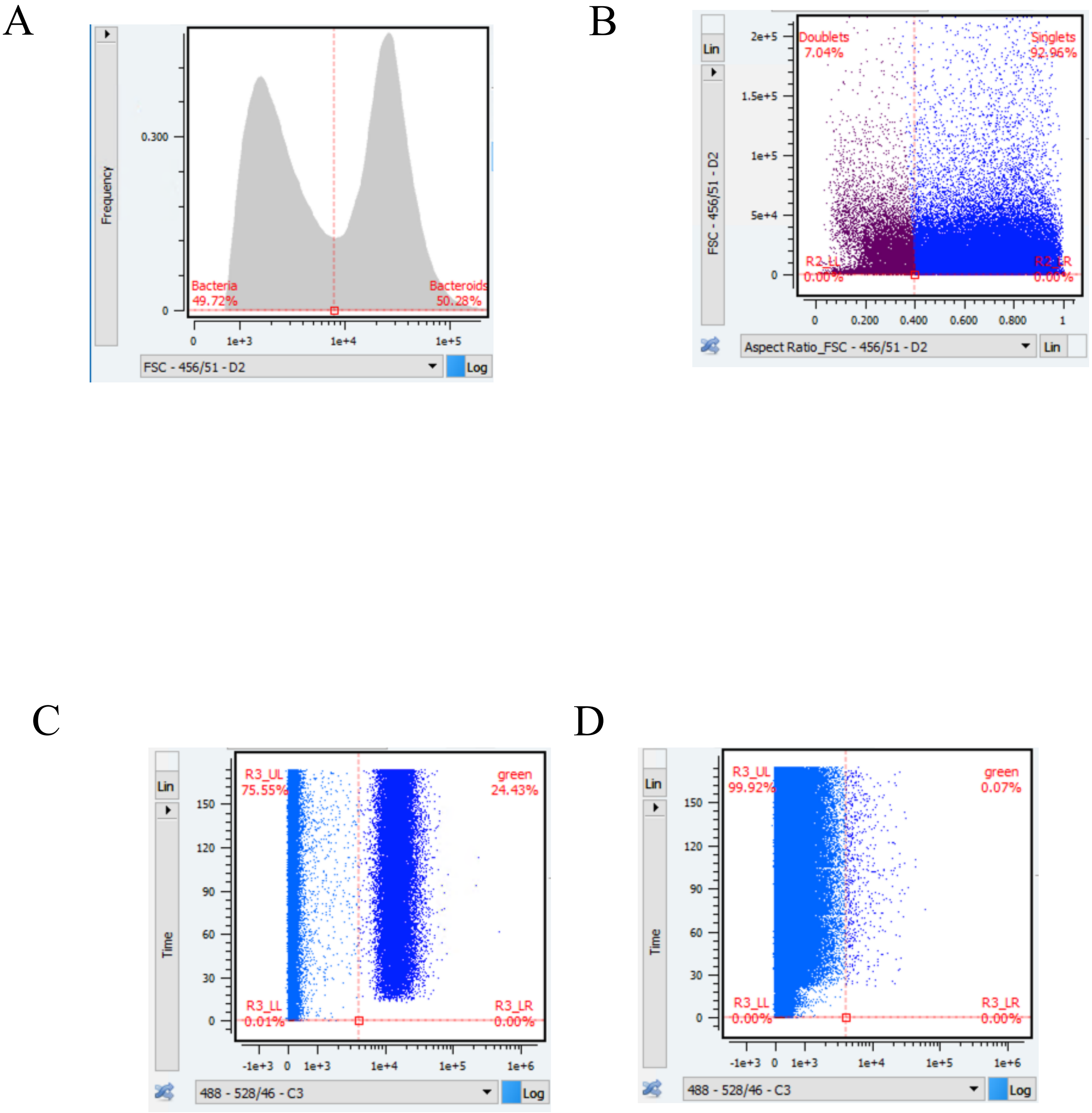
A
Frequency
0.300
0
Bacteria
49.72%
Bacteroids
50.28%
0
1e+3
1e+4
1e+5
FSC - 456/51 - D2
Log
B
Lin
2e+5
1.5e+5
1e+5
5e+4
0
FSC - 456/51 - D2
Doublets
7.04%
Singlets
92.96%
R2_LL
0.00%
R2_LR
0.00%
0
0.200
0.400
0.600
0.800
1
Aspect Ratio_FSC - 456/51 - D2
Lin
C
Lin
150
120
90
60
30
0
Time
R3_UL
75.55%
green
24.43%
R3_LL
0.01%
R3_LR
0.00%
-1e+3
0
1e+3
1e+4
1e+5
1e+6
488 - 528/46 - C3
Log
D
Lin
150
120
90
60
30
0
Time
R3_UL
99.92%
green
0.07%
R3_LL
0.00%
R3_LR
0.00%
-1e+3
0
1e+3
1e+4
1e+5
1e+6
488 - 528/46 - C3
Log

E

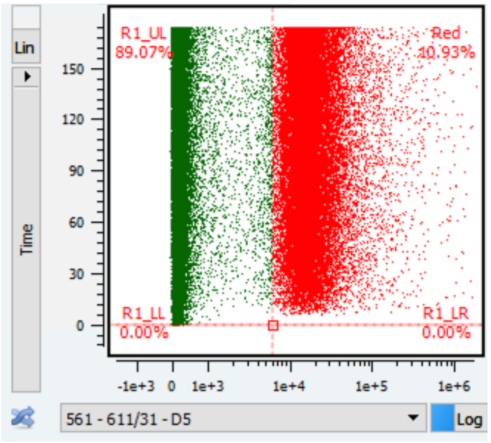
Lin
Time
150
120
90
60
30
0
R1_UL
89.07%
Red
10.93%
R1_LL
0.00%
R1_LR
0.00%
-1e+3
0
1e+3
1e+4
1e+5
1e+6
561 - 611/31 - D5
Log

F

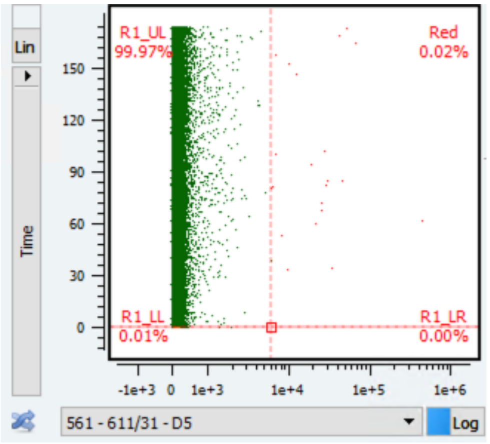
Lin
Time
150
120
90
60
30
0
R1_UL
99.97%
Red
0.02%
R1_LL
0.01%
R1_LR
0.00%
-1e+3
0
1e+3
1e+4
1e+5
1e+6
561 - 611/31 - D5
Log

Fig. S2 Formulae for back transformation for outputs of analyzes of $\log_{10}$ transformed data

$$E = 10^{A} - 10^{(A + B)}$$

$$SE = (10^{A} - 10^{(A + B)}) - (10^{A} - 10^{(A + B + C)})$$

Where:
E is the back transformed estimate difference between the two means

A is the $\log_{10}$ transformed estimate of the mean of the focal population

B is the estimate difference between A and the $\log_{10}$ transformed estimate of the comparison population

SE is the back transformed standard error of the estimate difference between the two $\log_{10}$ transformed means

Fig. S3 Confocal images of single occupant nodule sections: Peas were inoculated with one of three combinations of strains: Fix$^+$ & Fix$^-$, Fix^{int} & Fix$^-$ and Fix$^+$ & Fix^{int}. Strains were isogenic apart from fluorescent tag and fixation ability. 100µm slices were taken from nodules picked after 28, 35 and 42 days post inoculation for imaging. Images were assessed for evidence of cell death. After 28 days there was limited evidence for a change to cell health between the nodules containing the effective (Orange) and the ineffective (Turquoise) strains. After 35 days all nodules containing the effective strain remained healthy while the ineffective containing nodules showed evidence of cell death. After 42 days all nodules regardless of occupant showed clear evidence of cell death.

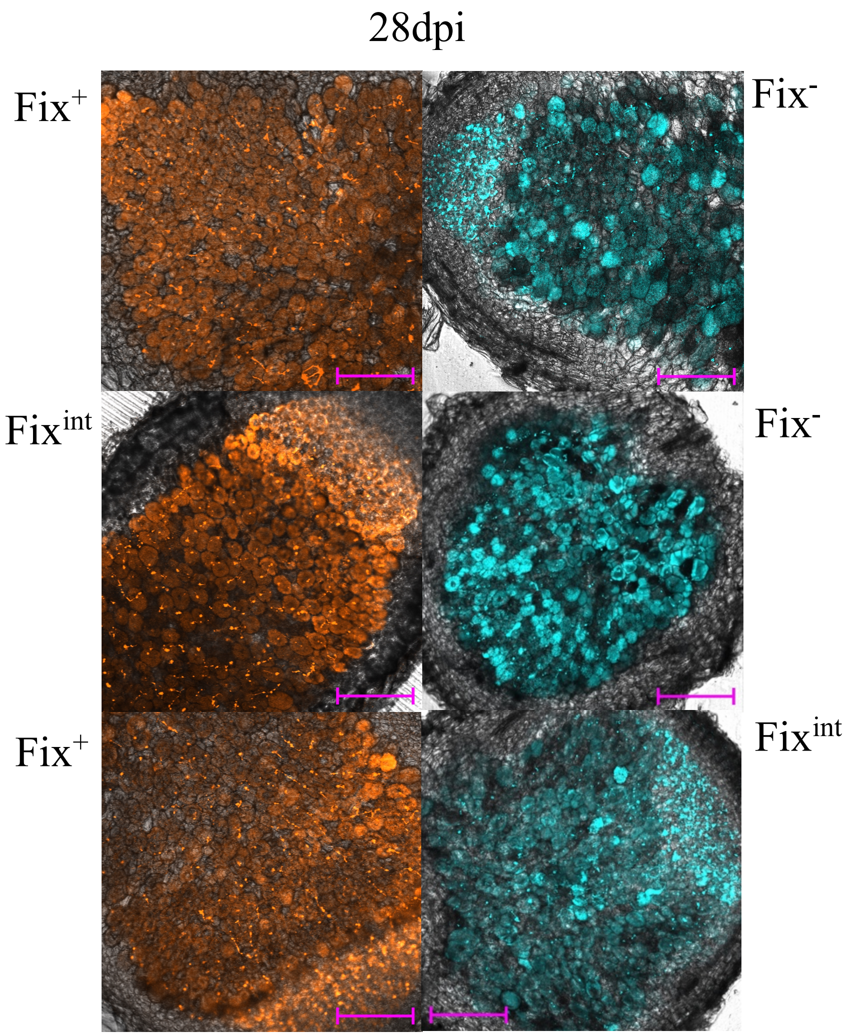

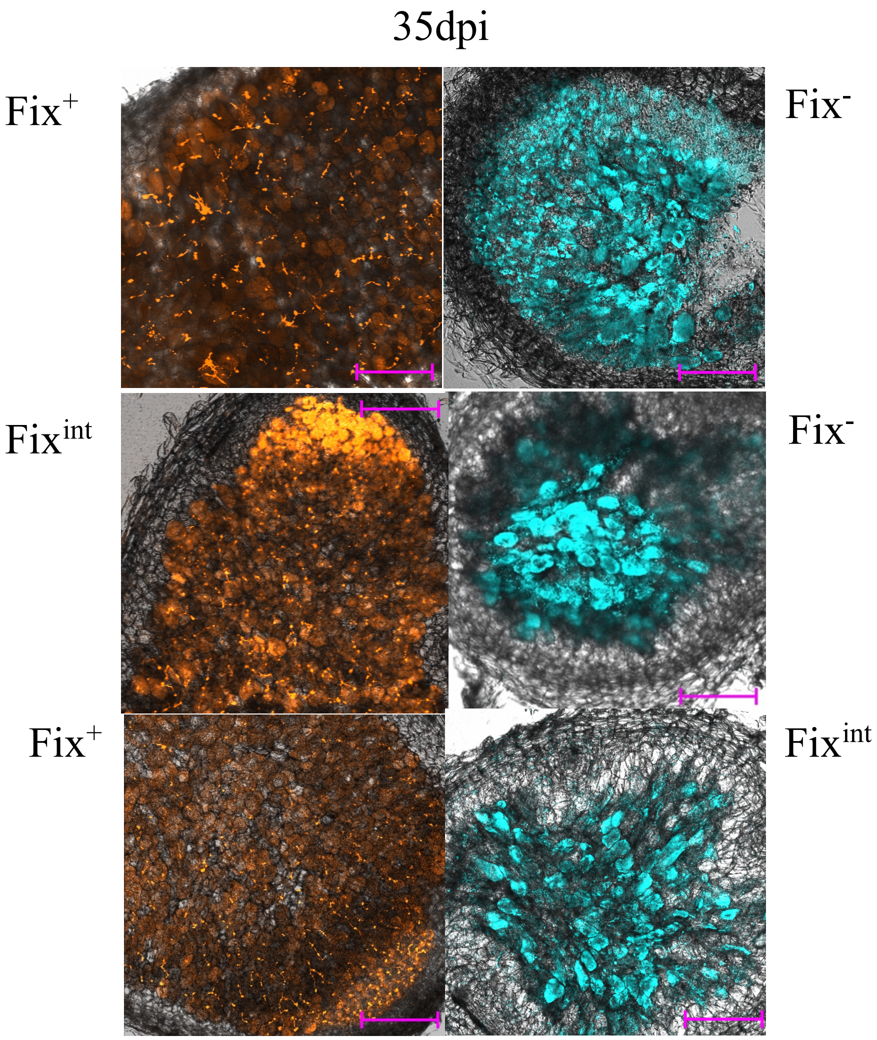
35dpi
Fix^{+}
Fix^{-}
Fix^{int}
Fix^{-}
Fix^{+}
Fix^{int}

42dpi

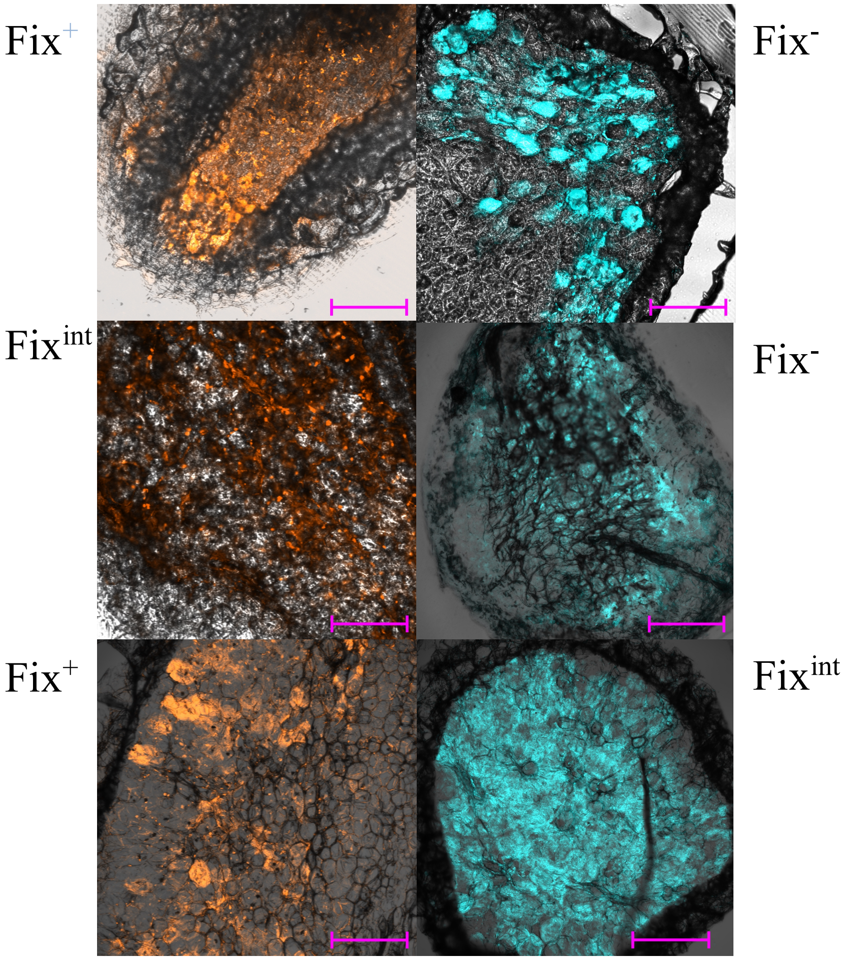

Supplement: erag191_Supplementary_Data [file erag191_supplementary_data.zip › JEXBOT316820-file003.pdf]
